# Social support and dairy products intake among adolescents: a study from Iran

**DOI:** 10.1186/s12889-015-2399-5

**Published:** 2015-10-22

**Authors:** Behjat Shokrvash, Leili Salehi, Maral Hariri Akbari, Mehrangiz Ebrahimi Mamagani, Saharnaz Nedjat, Mohammad Asghari, Freshteh Majlessi, Ali Montazeri

**Affiliations:** Department of Health Education and Promotion, School of Health, Tabriz University of Medical Sciences, Tabriz, Iran; Department of Health Education, School of Public Health, Alborz University of Medical Sciences, Karaj, Iran; Department of nutrition and food science, Danshvaran University, Bakmisheh, Ellaheie, Tabriz, Iran; Department of Community Nutrition, Tabriz University of Medical Sciences, Tabriz, Iran; Department of Epidemiology and Biostatistics, School of Public Health, Tehran University of Medical Sciences, Tehran, Iran; Knowledge Utilization Research Center, Tehran University of Medical Sciences, Tehran, Iran; Department of Statistics and Epidemiology, School of Health, Tabriz University of Medical Sciences, Tabriz, Iran; Department of Health Education and Promotion, School of Public Health, Tehran University of Medical Sciences, Tehran, Iran; Mental Health Research Group, Health Metrics Research Center, Iranian Institute for Health Sciences Research, ACECR, Tehran, Iran; Faculty of Humanity Sciences, University of Science & Culture, ACECR, Tehran, Iran

## Abstract

**Background:**

Adequate daily milk and dairy products intake seems to an important for adolescents’ health. This study aimed to identify the high-risk group adolescents who did not meet the recommended daily serving milk and dairy products and indeed to find out associated factors relating to their nutrition behaviors.

**Methods:**

This cross sectional study was carried out on 7th grade students, in Tabriz, East Azerbaijan province, Iran. An anonymous self–administrated questionnaire including items on perceived social support, physical activity, and sedentary behaviors was administered. In addition a valid food frequency questionnaire (FFQ) measuring daily milk products serving intake as a main outcome measure was completed for each respondent. Logistic regression analysis was applied to examine the association between milk and dairy products consumption and independents variables.

**Results:**

In all 402 students (51.5 % female) participated in the study. The mean age of students was 12.9 (SD = 0.49) years. The average daily intake of milk and dairy products was 1.64 (SD = 0.78) servings per day. Overall 14.2 % of adolescents (18.8 % of girls, and 9.2 % of boys, *p* = 0.006) reported consumption of the recommended daily milk and dairy products serving per day. The results indicated that gender boys (OR for boys = 2.41, 95 % CI = 1.25–4.67), mother age (OR for age group 40–55 years = 2.52, 95 % CI = 1.18–5.38), poor perceived emotional family support, (OR = 1.10, 95 % CI = 1.05–3.61), and poor perceived practical family support (OR = 2.04, 95 % CI = 1.18–4.17) were the most significant contributing factors to low level milk and dairy products intake in adolescents.

**Conclusion:**

The findings indicated that adolescents did not take the recommended daily amount of milk and dairy products and this appeared to be strongly related to low perceived family support. To achieve the recommended daily milk and dairy products serving consumption, family involvements in any programs that specifically address emotional and practical support for promoting daily milk and dairy products intake among adolescents are suggested.

## Background

Consumption of milk and dairy products is associated with numerous health benefits and play an important role in healthy lifestyle through the lifespan [1–3]. Milk and dairy products, because of their essential micronutrients content, have synergic effects on strengthening skeletal system [2, 3], enhancing body fitness and performance [4, 5], and preventing some medical conditions such as high blood pressure [6] and colon cancer [7] in youth and adulthood. However, despite health benefits, there is evidence indicating that some adolescents could not meet the recommended serving size of milk and dairy intake worldwide [8–14], while poor nutrient snacks consumption [10–12, 15], with assumed adverse health outcomes are increasing among adolescents [16, 17].

Several different reasons were suggested for inadequate and insufficient dairy products consumption among adolescents. Also it has been shown that the pattern and predictors of milk and dairy products consumption might differ in different adolescent populations [12, 14, 18]. Some attributing factors have a major role on their multi component eating behaviors [12], while other important factors such as psychosocial parameters and some unhealthy behaviors [12, 14, 19] have not been documented consistently [20, 21].

Iran has a young population (more than 50 % are aged less than 29 years). Despite such a high proportion of young population, there is limited evidence regarding the adolescents’ consumption of healthy diet. A national investigation studying a representative sample of 21,111 school students aged 6–18 years found that fruit and vegetables, dairy products and snacks (salty, fatty or sweet) had a similar consumption frequency of approximately twice a day [22]. Similarly an investigation from Tehran, the capital of Iran, studying 7669 adolescents (4070 boys and 3599 girls) revealed that although 82 % of girls and 75 % of boys had good nutritional knowledge, only 25 % of boys and 15 % of girls had good nutritional practice [23]. Yet, investigations on milk and diary products intake among Iranian adolescents are neglected. Therefore, the current study was designed to identify factors that are contributing to milk and dairy products intake. Also we were interested in assessing gender differences in dairy products intake. It was hoped that the findings from the study would add to the existing literature on the topic and provide essential information in developing tailored interventions for adolescent dairy products eating behavior. The results would be communicated to the health promotion office in ministry of health.

## Methods

### Participants

The participants were student adolescents studying at 7^th^ grade in Tabriz, center of East Azerbaijan province, Iran. There were 183 schools out of which 4 government schools were randomly selected (two boy and two girl schools). We surveyed all students in the 7^th^ grade of these four schools, where a free cup of milk was distributed among students every day. Sample size was estimated based on the prevalence of Iranian adolescent physical activity [24]. It was estimated that 88 students from each school (boys = 176, girls = 176, total = 352) would provide an enough sample size for comparing gender differences. A study with such a sample size would have a power of 80 % at 5 % significance level. However since we surveyed all the students in 7^th^ grade of these four schools, the actual sample size in this study was 402. Adolescents with any history of food allergy, under diet therapy and/or nutrition advisory, and those who had not completed the questionnaire were excluded.

### Questionnaires

We used several questionnaires to collect data. All measures underwent preliminary psychometric evaluations. Forty-five male and female students participated in a pilot study. To test reliability the internal consistency of the questionnaires were measured using the Cronbach’s alpha coefficient. Stability was assessed using the intraclass correlation coefficient (ICC) with a two-weeks interval. Content and face validity were performed to insure that students understand questions and were in-ease in responding to the questionnaires. The Cronbach’s alpha coefficient and the ICC for each measure are indicated as we describe the measures:

1. A demographic questionnaire included recording of information on age, gender, parental information (mothers’ age, mothers’ employment, parental education, and marital status) and socio economic status.
2. We used the Family Affluence Scale (FAS) in order to identify family socio-economic status. This is a suitable instrument for indicating socio-economic status in developing countries [25]. The scale consists of five different properties: family car ownership (0, 1, 2, 3 or more), computer and laptop ownership (0, 1, 2, 3 or more), number of rooms excluding kitchen and bathrooms (0, 1, 2, 3 or more), number of telephones (0, 1, 2, 3 or more), and having unshared bedroom (no = 0, yes = 1). Adolescents were asked to identify the number of owned items. Then, the overall scores were calculated by summing up the possible responses, giving a score ranging from 0 to 13. Accordingly the FAS score was categorized into three levels: low 0–4, intermediate 5–8, and high 9–13 (Cronbach’s a coefficient = 0.88, ICC = 0.80).
3. Self-efficacy questionnaire that consisted of 8 items derived from Watson et al. instrument [26]. Participants were asked if they were willing to increase the level of their daily serving milk and dairy products consumption or reduce unhealthy food and how confident they were to do so. The responding rate on the perceived self-efficacy employing on a 5-point Likert scale (very unsure = 1 to very sure = 5) provided a possible score ranging from 8 to 45. (Cronbach’s alpha coefficient = 0.86, ICC = 0.81).
4. Family support specific to healthy eating consisted of a 16-item questionnaire containing questions about perceived informational family support (PIFS-3 items), perceived emotional family support (PEFS-3 items), and perceived practical family support specific to healthy eating (PPFS-11 items). The questions were derived from the very well known questionnaires developed by Sallis [27] and Stanton [28]. Participants were asked how often their mother would advised, told, and gave them information about dairy consumption servings (informational support); how often their mother would encourage them to drink milk and to eat dairy foods, how often their mother would admire them during milk and dairy intake, how often their mother would watch their consumption (emotional support); how often their mother would share drinking and eating milk and dairy products with them, how often their mother would provide milk and dairy products so they could consume whenever they wanted to, how often their mother would provide milk and dairy products as snack for them to take to school (practical support). The response category on perceived support employing a 5-point Likert scale (never = 1; rarely = 2; often = 3; sometimes/usually = 4; always = 5) provided a possible score ranging from 3 to15 for informational support, and for emotional support, and 11 to 55 for practical support (Cronbach’s alpha ranged from 0.76 to 0.88 and ICC ranged from 0.73 to 0.79).
5. To assess frequency and duration of physical activity levels, we used the well-validated modified version of the Adolescent Physical Activity and Recall Questionnaire (APARQ) [29]. The questionnaire consisted of a number of items on common activities and games that adapted from previous study except that we did not separate organized and/ or non-organized activities on the week before [24]. Then, responses were categorized into light, moderate, and vigorous activities according to the estimated rate of energy expenditure (METs) for each activity [24].
6. The food frequency questionnaire (FFQ) including 118 food items from three main food groups and subgroups (fruit, vegetables, milk and dairy products, salty food, fast food, sweet food) was used to estimate eating behaviors. We used the FFQ that was validated for Iranian population [30]. For the purpose of this study we only used the data related to milk and dairy products and sweet food. Milk and dairy product items included all types of milks (whole, low fat, skim, cocoa and chocolate), Yogurt (plain and whole, concentrated and creamy), Doogh (yogurt drink), Kashk (dried or thick yogurt), and cheese (plain and creamy). Sweet food was included because of the high intake of the sweet food and its significant effects as unhealthy foods. The participants were asked to recall the frequency of foods they consumed during previous week. They were also requested to estimate the amounts of each food item according to the determined domestic scales [30], which adapted based on the standard portion sizes [8]. The possible responses were ranged from never to 3 or more servings per day and per week. All responses were recoded into daily consumption (never = zero serving, smaller than one serving, one serving, 2 servings, 3 and/or more servings per day). The average daily consumption for each food group was computed by summing up the items of food in each group indicated by adolescents (Cronbach’s alpha coefficient ranged from 0.78 to 0.87 and ICC ranged from 0.74 to 0.78 respectively).

### Procedure

This cross sectional study of eating behaviors was carried out among 7^th^ grade adolescent students in Tabriz, Iran. After obtaining written consent from authorities, schools administrators and one of the parents, the timetable for data collection was provided. Adolescents completed the questionnaires during lesson times on the third week of starting academic year in October 2010. The main investigator (BS) administered the survey questionnaire and was available to answer the possible questions. An equal time of 45 min was considered for all adolescents to fill in the questionnaires.

### Statistical analysis

Descriptive statistics including frequency, percentage, mean, and standard deviations were used to represent the data. Both univariate and multivariate logistic regression analysis were performed to examine the association between dependent variable (milk and dairy products consumption) and independent variables including age, gender, mother age and employment, parental education years, perceived family support, self-efficacy, physical activity levels, sedentary activity and sweet food. To perform logistic regression analysis, the dependent variable (milk and dairy products consumption) was categorized into two levels: equal or greater than 3 servings per day (desirable outcome) and less than 3 servings per day (undesirable outcome) [8]. The independent variables such as physical activity and sedentary behaviors were categorized into two levels: attained the WHO guideline (equal or greater than 60 min/day, and equal or less than120 min/day) and did not attain the guideline (less than 60 min/day for physical activity and greater than 120 min/day for sedentary behaviors) [31]. All analyses were performed for entire sample and separately for girls and boys. The data were analyzed using SPSS statistics software version 11.5 (SPSS Inc, IL. Chicago, USA).

### Ethics

The ethics committee of Tehran University of Medical Sciences approved the study. An informed written consent was obtained from all adolescents. In addition consent was obtained from parents. Adolescents could choose to withdraw from the study any time before or during the completion of the questionnaires.

## Results

### Participants

Overall 402 students completed the questionnaires. The mean age of participants was 12.9 (SD = 0.49) years ranging from 12 to 15, and 51.5 % were female. There were significant differences between boys and girls in some characteristics including self-efficacy, mother education and employment status. The characteristics of the study sample are shown in Table 1.

**Table 1:** The characteristics of the study sample

	All (*n* = 402)	Girls (*n* = 207)	Boys (*n* = 195)	*P*-value
	No. (%)	No. (%)	No. (%)	
Age (yrs)				0.452^*^
≤12	65 (16.1)	35 (16.9)	30 (15.4)	
13	301 (74.9)	157 (75.8)	144 (73.8)	
≥14	36 (9)	15 (7.2)	21 (1.08)	
Mean (SD)	12.93 (0.49)	12.90 (0.47)	12.95 (0.50)	0.302^**^
Mother’s age (yrs)				0.163^*^
20–34	120 (29.9)	61 (29.5)	59 (30.3)	
35–39	143 (35.6)	82 (39.6)	61 (31.3)	
40–55	139 (34.6)	64 (30.9)	75 (38.5)	
Mean (SD)	37.4 (5.13)	36.97 (4.50)	37.83 (5.69)	0.091^**^
Mother’s employment (No., %)				<0.001^*^
Housewife	341 (84.8)	161 (77.8)	180 (92.3)	
Employed	61 (15.2)	46 (22.2)	15 (7.7)	
Mother’s education (yrs)				0.020^*^
0–12	352 (87.6)	174 (84.1)	178 (91.3)	
>12	50 (12.4)	33 (15.9)	17 (8.7)	
Mean (SD)	10.36 (3.39)	10.99 (3.03)	9.89 (3.62)	<0.001^**^
Father’s education (yrs)				
0–12	320 (79.6)	156 (75.4)	164 (84.1)	
>12	82 (20.4)	51 (24.6)	31 (15.9)	
Mean (SD)	10.89 (3.66)	11.48 (3.31)	10.27 (3.98)	0.001^**^
Parent marital status (No., %)				0.052^*^
Married	385 (95.8)	202 (97.6)	183 (93.8)	
Widowed	17 (4.2)	5 (2.4)	12 (6.2)	
FAS				0.811^*^
High (9–13)	32 (8)	19 (9.2)	13 (6.7)	
Intermediate (5–8)	309 (76.8)	163 (78.7)	146 (74.9)	
Low (0–4)	61 (15.2)	25 (12.1)	36 (18.5)	
Physical activity (minutes/day)				
Mean (SD)	44.64 (23.24)	38.77 (19.94)	50.87 (24.88)	<0.001^**^
≥60	89 (22.1)	35 (16.90)	54 (27.7)	
Sedentary activity (minutes/day)				
Mean (SD)	192.83 (89.23)	170.26 (89.20	218.25 (103.34	<0.001^**^
≤120	139 (34.6)	93 (44.9)	46 (23.6)	
Self-efficacy score				
Mean (SD)	29.01 (7.09)	29.04 (7.25)	28.97 (7.04)	0.923^**^
Score range	8–40	8–40	8–40	
PIFS score				
Mean (SD)	18.55 (5.62)	18.68 (6.64)	18.4 (4.28)	0.611^**^
Score range	3–15	3–15	3–15	
PEFS score				
Mean (SD)	9.31 (3.2)	9.35 (3.31)	9.25 (3.15)	0.754^**^
Score range	3–15	3–15	3–15	
PPFS score				
Mean (SD)	25.46 (5.50)	26.61 (5.62)	24.25 (5.07)	<0.001^**^
Score range	11–55	11–55	11–55	

^*^Derived from chi-square, ^**^Derived from *t*-test

FAS: Family affluence scale

PIFS: Perceived informational family support

PEFS: Perceived emotional family support

PPFS: Perceived practical family support

### Daily milk and dairy products intake among adolescents

The average daily intake of milk and other dairy products was 1.64 (SD = 0.78) servings. Only 14.2 % of adolescents reported meeting the recommended daily milk and dairy products serving consumption. The average daily intake of milk and other dairy products among girls was1.64 (SD = 0.86) servings per day. This figure for boys was 1.63 (SD = 0.70) servings per day. Compared to boys, a higher percentage of girls (18.8 % versus 9.2 %, *p* = 0.006) reported meeting the recommended 3 servings milk and dairy products consumption (Table 2).

**Table 2:** Distribution and comparison of daily and recommended intake of milk and dairy products by gender

	All (*n* = 402)	Girls (*n* = 207)	Boys (*n* = 195)	*P*-value^*^
Milk and dairy products intake^a^				
Mean (SD)	1.64 (0.78)	1.64 (0.86)	1.63 (0.70)	0.833
Frequency (%) for recommended intake of daily milk and dairy products (≥3 serving/d)	57 (14.2)	39 (18.8)	18 (9.2)	0.006
Sweet food intake^b^				
Mean (SD)	3.13 (1.22)	3.01 (1.27)	3.23 (1.11)	0.064

^*^Derived from *t*-test

^a^One serving of the milk and dairy products is equal to a 200^cc^cup of all types of milks (whole, low fat, skim, cocoa and chocolate), yogurt (plain and whole), yogurt (concentrated and creamy), 2 cup of yogurt drink, cheese (plain and creamy), and one tablespoon dried or thick yogurt, and half of cup of ice cream (plain and high fat traditional)

^b^All sweet foods were measured by the domestic scales that adapted for standard portion sizes [8]. One serving of each sweet beverages, soft drinks, beer (non-alcoholic), syrup is equal to a cup; simple sugar a teaspoon; honey and jams a tablespoon; plain cake, chocolates, pastries (non-crème and creamy) a slice; cubed sugar, yazdi cake, all biscuits other than those made from whole grain, gaz, sohan a number; crème caramel, domestic halvah a tablespoon and non-domestic halvah a pocket (adapted from [30])

### Predictors of milk and dairy products intake

The results obtained from multiple logistic regression analysis indicated that gender (OR for boys =2.28, 95 % CI = 1.25–4.15), mother age (OR for age 40–55 years = 2.52, 95 % CI = 1.18–5.38), poor perceived emotional family support (OR = 1.10, 95 % CI = 1.05–3.61), and poor perceived practical family support (OR = 2.04, 95 % CI = 1.18–4.17) were the most significant contributing factors to low level milk and diary consumption in adolescents. Other variables such as physical activity, and sedentary behaviors did not show any significant results. The results are presented in Table 3 for whole sample. When the analysis was performed for girls and boys separately, none of the variables showed significant relationship with low level milk and dairy products consumption in boys (Table 4), while for girls (Table 5) we found a significant association between poor milk and diary intakes and low perceived practical family support (OR = 1.08, 95 % CI = 1.04–2.04, *P* = 0.036).

**Table 3:** The results obtained from logistic regression analysis for low serving milk and dairy products consumption among adolescents (*n* = 402)

	Unadjusted OR (95 % CI)	*P*-value	Adjusted OR (95 % CI)^a^	*P*-value
Gender				
Girl	1.0 (ref)		1.0 (ref)	
Boy	2.28 (1.25–4.15)	0.007	2.41 (1.25–4.67)	0.009
Age (yrs)				
≤ 12	1.0 (ref)		1.0 (ref)	
13	1.10 (0.50–2.24)	0.874	1.11 (0.52–2.41)	0.784
≥ 14	2.01 (0.51–7.79)	0.318	2.04 (0.47–7.98)	0.357
Mother’s age (yrs)				
20–34	1.0 (ref)		1.0 (ref)	
35–39	1.24 (0.65–2.36)	0.524	1.41 (0.72–2.75)	0.316
40–55	2.17 (1.04–4.54)	0.038	2.52 (1.18–5.38)	0.017
Mother’s employment				
Housewife	1.0 (ref)		1.0 (ref)	
Employed	1.11 (0.49–2.48)	0.796	1.23 (0.48–3.16)	0.656
Mother’s education (yrs)				
> 12	1. 0 (ref)		1. 0 (ref)	
0–12	1.01 (0.33–1.98)	0.637	1.04 (0.21–1.93)	0.658
Father’s education (yrs)				
> 12	1. 0 (ref)		1. 0 (ref)	
0–12	2.03 (0.97–3.43)	0.059	1.04 (0.27–1.07)	0.078
FAS				
High	1.0 (ref)		1.0 (ref)	
Intermediate	1.10 (0.91–2.60)	0.788	1.10 (0.16–2.67)	0.572
Low	1.10 (0.23–2.92)	0.766	1.11 (0.24–2.61)	0.706
Self efficacy score	1.10 (0.95–1.12)	0.421	1.10 (0.94–1.34)	0.390
PIFS score	1.11 (0.95–1.22)	0.916	1.03 (0.95–1.30)	0.439
PEFS score	1.11 (0.98–2.28)	0.065	1.10 (1.05–3.61)	0.034
PPFS score	2.45 (1.89–5.90)	0.037	2.04 (1.18–4.17)	0.025
Physical activity (minutes/day)	1.02 (0.34–1.48)	0.369	1.11 (0.36–1.66)	0.517
Sedentary activity (minutes/day)	1.34 (0.75–2.37)	0.324	1.10 (0.60–2.30)	0.762
Sweet food intake	1.02 (0.75–1.16)	0.493	1.11 (0.69–1.13)	0.332

^a^Adjusted for adolescents’ age and gender, mother’s age and employment, parental education, FAS, self-efficacy, PIFS, PEFS, PPFS, physical activity, sedentary activity, and sweet food intake

FAS: Family affluence scale

PIFS: Perceived informational family support

PEFS: Perceived emotional family support

PPFS: Perceived practical family support

**Table 4:** The results obtained from logistic regression analysis for low serving milk and dairy products consumption among boys (*n* = 195)

	Unadjusted OR (95 % CI)	*P*-value	Adjusted OR (95 % CI)^a^	*P*-value
Age (yrs)				
≤ 12	1.0 (ref)		1.0 (ref)	
13	1.03 (0.31–3.84)	0.963	1.56 (0.92–5.79)	0.643
≥ 14	2.22 (0.22–22.97)	0.503	4.53 (0.90–16.76)	0.879
Mother’s age (yrs)				
20–34	1.0 (ref)		1.0 (ref)	
35–39	3.11 (0.74–8.84)	0.136	2.30 (0.64–8.09)	0.534
40–55	3.02 (0.79–7.97)	0.116	2.11 (0.05–7.64)	0.368
Mother’s employment				
Housewife	1.0 (ref)		1.0 (ref)	
Employed	2.34 (0.26–11.79)	0.723	3.40 (0.89–14.34)	0.589
Mother’s education (yrs)				
> 12	1.0 (ref)		1.0 (ref)	
0–12	1.10 (0.85–4.74)	0.621	1.23 (0.29–3.76)	0.769
Father’s education (yrs)				
> 12	1.0 (ref)		1.0 (ref)	
0–12	1.10 (0.28–3.92)	0.925	1.08 (0.98–5.78)	0.986
FAS				
High	1.0 (ref)		1.0 (ref)	
Intermediate	1.10 (0.28–3.93)	0.925	1.04 (0.19–3.63)	0.813
Low	1.10 (0.07–4.74)	0.621	1.01 (0.02–8.74)	0.064
Self efficacy score	1.11 (0.93–1.14)	0.931	1.11 (0.93–1.28)	0.074
PIFS score	1.11 (0.96–1.25)	0.171	1.11 (0.95–1.34)	0.207
PEFS score	1.12 (0.95–1.32)	0.080	1.10 (0.88–1.34)	0.410
PPFS score	1.11 (0.84–1.20)	0.413	1.05 (0.85–1.11)	0.321
Physical activity (minutes/day)	1.37 (0.43–4.28)	0.768	1.09 (0.76–6.34)	0.658
Sedentary activity (minutes/day)	1.71 (0.60–4.85)	0.890	2.43 (0.74–6.58)	0.785
Sweet food intake	1.04 (0.54–1.32)	0.341	1.34 (0.86–3.25)	0.654

^a^Adjusted for adolescents’ age, mother’s age and employment, parental education, FAS, self-efficacy, PIFS, PEFS, PPFS, physical activity, sedentary activity, and sweet food intake

FAS: Family affluence scale

PIFS: Perceived informational family support

PEFS: Perceived emotional family support

PPFS: Perceived practical family support

**Table 5:** The results obtained from logistic regression analysis for low serving milk and dairy products consumption among girls (*n* = 207)

	Unadjusted OR (95 % CI)	*P*-value	Adjusted OR (95 % CI)^a^	*P*-value
Age (yrs)				
≤ 12	1.0 (ref)		1.0 (ref)	
13	1.11 (0.42–2.65)	0.904	1.14 (0.45–3.40)	0.741
≥ 14	2.03 (0.29–8.93)	0.576	2.10 (0.24–9.80)	0.643
Mother’s age (yrs)				
20–34	1. 0 (ref)		1.0 (ref)	
35–39	1.10 (0.43–2.15)	0.927	1.14 (0.19–3.45)	0.987
40–55	2.11 (0.73–4.96)	0.192	3.45 (0.95–8.34)	0.564
Mother’s employment				
Housewife	1.0 (ref)		1.0 (ref)	
Employed	1.4 (0.56–3.41)	0.419	1.45 (0.18–6.87)	0.543
Mother’s education (yrs)				
> 12	1.0 (ref)		1.0 (ref)	
0–12	1.01 (0.33–1.98)	0.477	1.34 (0.89–4.75)	0.956
Father’s education (yrs)				
> 12	1.0 (ref)		1.0 (ref)	
0–12	2.03 (0.96–3.43)	0.060	2.11 (0.98–3.59)	0.860
FAS				
High	1.0 (ref)		1.0 (ref)	
Intermediate	1.23 (0.04–3.98)	0.727	1.02 (0.17–3.95)	0.802
Low	1.04 (0.20–3.55)	0.817	1.11 (0.30–3.84)	0.911
Self efficacy score	1.11 (0.93–1.13)	0.363	1.10 (0.94–1.21)	0.367
PIFS score	1.11 (0.93–1.14)	0.596	1.03 (0.96–1.10)	0.445
PEFS score	1.05 (0.85–1.41)	0.315	102 (0.88–1.58)	0.419
PPFS score	1.10 (0.89–1.33)	0.228	1.08 (1.04–2.04)	0.036
Physical activity (minutes/day)	1.15 (0.44–2.98)	0.690	2.43 (0.98–9.23)	0.854
Sedentary activity (minutes/day)	1.40 (0.46–1.88)	0.419	1.64 (0.98–2.56)	0.677
Sweet food intake	1.10 (0.62–1.21)	0.786	1.09 (0.98–3.45)	0.902

^a^Adjusted for adolescents’ age, mother’s age and employment, parental education, FAS, self-efficacy, PIFS, PEFS, PPFS, physical activity, sedentary activity, and sweet food intake

FAS: Family affluence scale

PIFS: Perceived informational family support

PEFS: Perceived emotional family support

PPFS: Perceived practical family support

## Discussion

The findings showed that average daily milk and dairy products consumption among boys and girls participated in the study was lower than the daily-recommended servings per day. In fact a high proportion of adolescents consumed milk and milk products less than the daily recommendations. Only 14.2 % of adolescents were recorded consuming optimal 3 and more serving milk and dairy products every day. Also when the average daily milk and dairy products intake was compared among boys and girls, we found that girls reported more daily consumption of milk and dairy products. While some investigators also reported similar findings [32, 33], some other reported different results [12, 19]. As such it seems that the observed differences might be due to difference in studying different populations and/or different age groups.

There were no significant associations between low-level physical activity, high-level sedentary behaviors, sweet food, and poor milk and dairy products intake among boys and girls while studies have shown that poor milk and dairy products intake was associated with poor physical activity and high sedentary behaviors [20, 33, 34]. It seems that there might be other reasons and mechanisms behind multi components eating behaviors among our study population regardless of their physical activity levels including the fact that our sample was derived from a transitional environment where people are moving from a traditional to a modern environment and thus adopting modern life styles [35].

We found that mothers’ age was one of the strong parental factors in taking low serving milk and dairy products among Iranian adolescents. The findings showed that with increase in mother’s age, the risk of less milk and dairy products intake increased among adolescents. Such an observation might be explained by the fact that perhaps older women had limited knowledge about adolescents’ nutrient requirements, especially on milk and dairy products intake, or there might be problems in mother and child relationships. In terms of the adverse role of family as a determining factor, it is argued that the daily consumption of milk and dairy products can be hampered through poor parental knowledge toward adolescent’s healthy eating needs [36, 37].

This study did not find any significant association between adolescent self-efficacy and lower milk products consumption [12, 38, 39]. Similarly a study found that interests toward healthy eating was not related to pre-adolescent (7^th^ grade students) self-efficacy, but strongly associated with 11^th^ grad student interests in healthy eating [40]. Milk and dairy product intake among adolescents at 7^th^ grade was not a personal, but a social issue. Poor perceived emotional and practical family support were the most significant contributing factors to low-level milk products consumption of adolescents. It was found that perceived family support had a significant contributing role to daily dairy products consumption among adolescents. In fact, when data were analyzed separately, low perceived practical support were found to be significant predictors of low milk and dairy products consumption even among the girls. Adolescents with low perceived practical family support were at risk of lacking in daily milk and dairy products intake. Numerous studies have shown the positive role of family as provider and role modeling [12, 20, 28]. This positive association shows that those adolescents who perceived practical support from mothers may be those who had more than 3 daily servings of milk and dairy products consumption. As shown in several studies, milk products consumption is primarily related to both family environment and social issues [35, 36, 41].

We did not find any significant association between low-level milk and dairy products intake by boys and personal and psychosocial predictors, while studies reported that parental education, tasty milk [14], home availability of milk, preference of milk taste, breakfast eating habits, higher socioeconomic status and social support were positive contributing factors to milk and dairy products intake [12]. However, it seems that other pertinent factors may influence on male adolescents dairy products intake that needs further investigation in a larger sample of adolescents with different age groups.

Family involvement to enhance milk and dairy products intake among adolescents can be possible through any family-centered program that specifically support adolescents emotionally and practically. Several strategies can be induced from family side to assist adolescents. The strategies may consist of encouraging the adolescents to take the milk and dairy products as much as possible, making the products available at home, preparing tasty snacks on preferable modes, and acting as a positive role model in consuming the milk products. Even there is evidence that frequent family meals might prevent poor quality diets among adolescents [42].

### Limitations

The reliance on self-reported food intake and perceived family support by adolescents are limitations. In addition we did not collect data on father, sibling and peers support. Additional research are needed to determine and compare the predictive values of the other potential social support sources including father, sibling and peers to better understand the influences of parental support on adolescent eating behavior and other health behaviors. We also suggest that future studies should include the personal and family misconception parameters about dairy foods. Finally we examined the association between milk and dairy products intakes with sweet foods while combined all sweet products together. Maybe it would be better to associate dairy consumption, particularly milk consumption with sweetened beverages consumption only, without combining sweet food together. Studies have shown that milk is often replaced by these beverages in adolescents.

## Conclusion

The findings of the current study that examined only milk and dairy products intake among adolescents indicated that both girls and boys did not meet the recommended daily milk and dairy products servings (3 serving per day). However, male students were at risk of getting less milk and dairy products. The results also showed that low perceived practical and emotional family support highly affected adolescent milk and dairy products intake regardless of their physical activity, sedentary behaviors and sweet food intake. To achieve the recommended daily milk and dairy products serving consumption, family involvements in any programs that specifically address emotional and practical support for promoting daily intake of milk and dairy products among adolescents are suggested.
